# The Era of Plant Breeding: Conventional Breeding to Genomics-assisted Breeding for Crop Improvement

**DOI:** 10.2174/1389202924666230517115912

**Published:** 2023-06-23

**Authors:** Thumadath Palayullaparambil Ajeesh Krishna, Duraipandiyan Veeramuthu, Theivanayagam Maharajan, Mariapackiam Soosaimanickam

**Affiliations:** 1 Division of Plant Biotechnology, Entomology Research Institute, Loyola College, Chennai, Tamil Nadu, India;; 2 Department of Advanced Zoology & Biotechnology, Loyola College, Nungambakkam, Chennai, 600034, India

**Keywords:** Crop improvement, plant breeding, conventional breeding, QTL, NGS, GS

## Abstract

Plant breeding has made a significant contribution to increasing agricultural production. Conventional breeding based on phenotypic selection is not effective for crop improvement. Because phenotype is considerably influenced by environmental factors, which will affect the selection of breeding materials for crop improvement. The past two decades have seen tremendous progress in plant breeding research. Especially the availability of high-throughput molecular markers followed by genomic-assisted approaches significantly contributed to advancing plant breeding. Integration of speed breeding with genomic and phenomic facilities allowed rapid quantitative trait loci (QTL)/gene identifications and ultimately accelerated crop improvement programs. The advances in sequencing technology helps to understand the genome organization of many crops and helped with genomic selection in crop breeding. Plant breeding has gradually changed from phenotype-to-genotype-based to genotype-to-phenotype-based selection. High-throughput phenomic platforms have played a significant role in the modern breeding program and are considered an essential part of precision breeding. In this review, we discuss the rapid advance in plant breeding technology for efficient crop improvements and provide details on various approaches/platforms that are helpful for crop improvement. This review will help researchers understand the recent developments in crop breeding and improvements.

## 1 INTRODUCTION

**Fig. (1):**
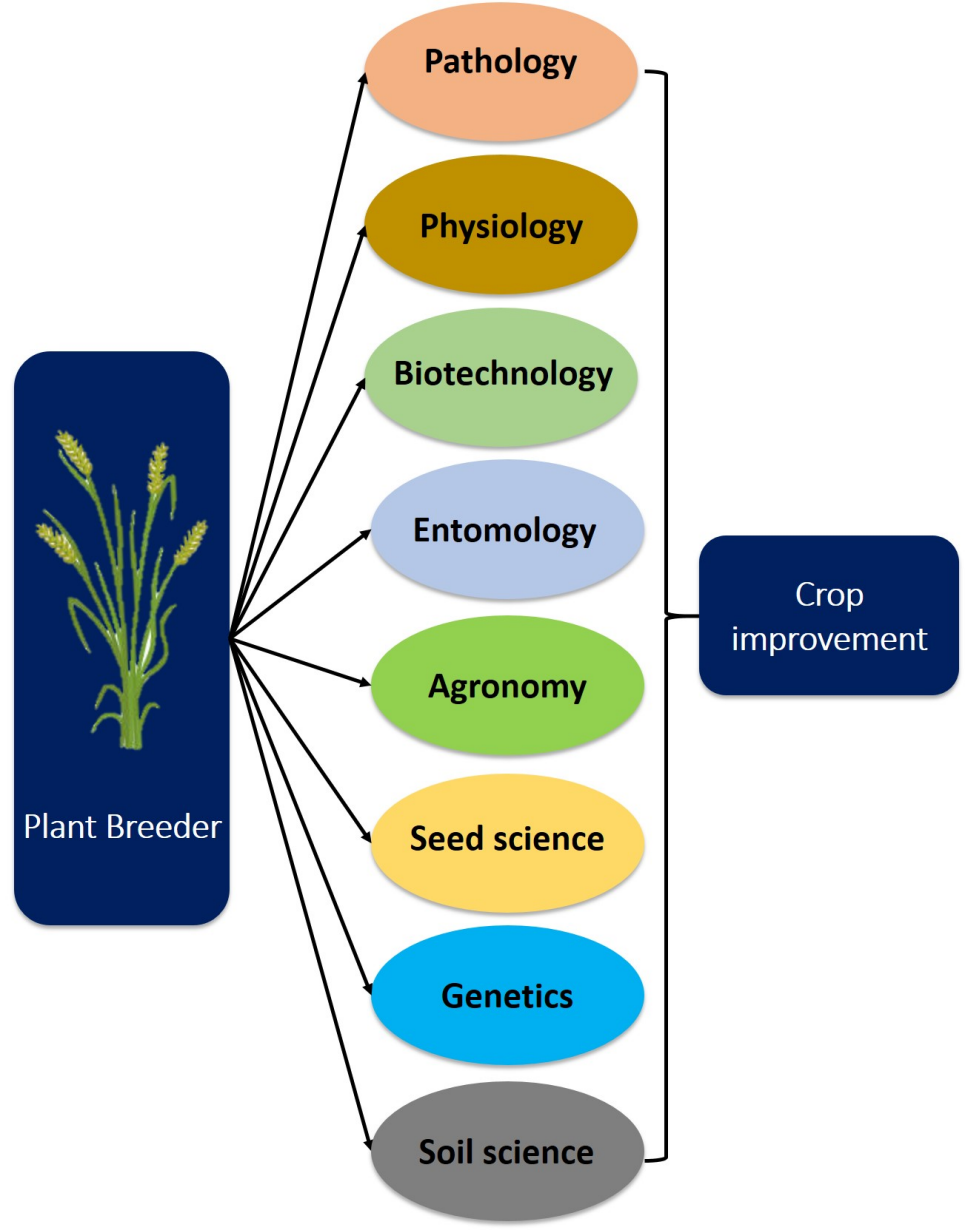
Various aspects of crop improvement. This flow chart indicates the role of plant breeders in crop improvement incorporating various traits.

Agriculture is the backbone of developing countries. Global climatic changes can influence agricultural production. The world population has been increasing daily; it may increase to 9.8 billion by 2050, leading to a global food demand [1]. Agricultural scientists are under tremendous pressure to raise agriculture productivity under the global climatic changes to feed the world population. Strengthening food security is possible only by developing sustainable crop varieties that adapt to global climatic changes [2]. The plant breeders focus on developing a better variety of desirable traits by modifying the genetic makeup of the crop. Plant breeding plays a crucial role in improving crop yield (Fig. **1**). Plant breeders contribute a lot to increase agriculture production worldwide.

Crop breeding is recombining desirable genes (related to crop yield) from different parents [3]. In the past, better crop selection was based on the natural variations (phenotypic traits) observed in the crop germplasm in a natural field. Phenotypic variations of crops are used for developing new varieties through conventional breeding. It has many drawbacks because environmental factors influence phenotypic characters, leading to the selection of poor breeding materials [4]. Also, it could lead to the narrowing of the gene pool of the germplasm and hamper the efficiency of crop improvement. Crop breeding based on the monitored recombination of genes of interest within the genome is crucial for efficient crop improvement. The progress in breeding technology, especially molecular marker-assisted selection (MAS), is very helpful and improves breeding efficiency [5]. The molecular markers are not interfered with by any environmental factors and are accurate during the selection, making MAS more attractive for breeding [6]. It helps track the genes responsible for tolerance within the crop genome and improves precision breeding.

Next-generation sequencing (NGS) technology has provided opportunities to carry out genome-assisted crop improvement [7-11]. The NGS technology offers the opportunity for genomic selection or genome-wide selection (GS) through genome-wide association studies (GAWS) or whole-genome association studies (WGAS). The genomic-assisted tools are helpful for rapidly selecting desirable traits from the huge germplasm, reducing breeding time, confirming interest in intermediate materials, and validating genes of interest in the gene pool [11-13]. The genomic approaches provide a strong foundation for efficient crop improvement. Advanced breeding technology can help sustainable agricultural production. It may also help to strengthen nutritional and food security under adverse climatic conditions in the future. In this review, we discuss the rapid advances in plant breeding technology for efficient crop improvement. We also provide details on conventional and genomic-assisted breeding (GAB) useful for crop improvement. We also provide details on various approaches/platforms helpful for crop breeding. Integrating advanced plant breeding techniques can rapidly identify valuable quantitative trait loci (QTL)/genes and ultimately speed up crop improvement programs.

## 2 SCOPE OF PLANT BREEDING

**Fig. (2):**
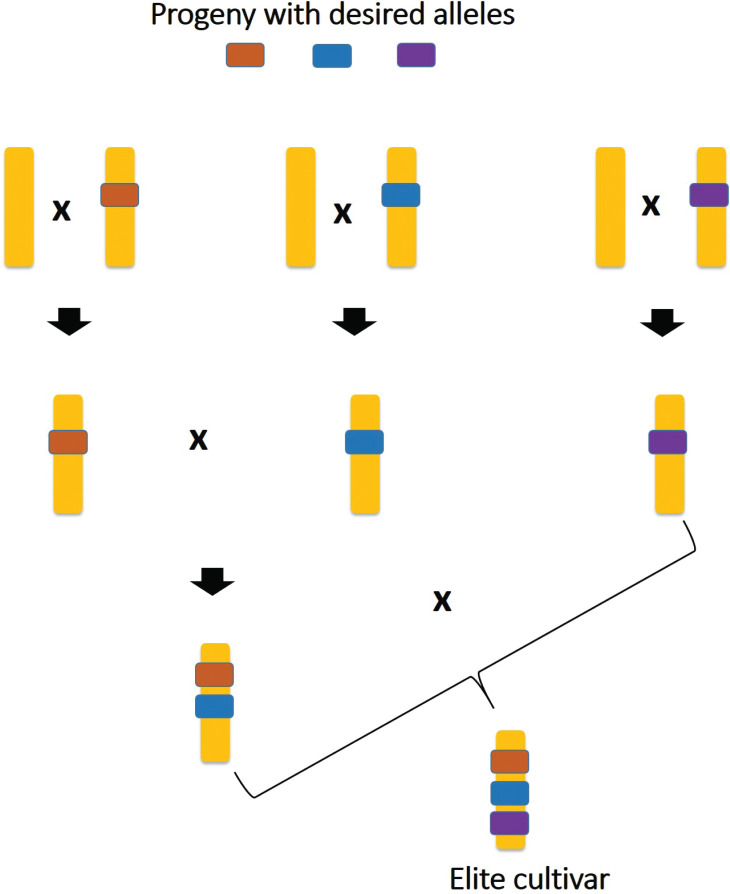
Strategies for developing new cultivar through breeding. Plant breeding provides an opportunity to integrate desirable traits and develop new crop cultivars with multiple genes for biotic and abiotic stress tolerance.

The world's food security depends upon sufficient agricultural production and access to food [14]. Plant breeding mainly focuses on developing improved crop varieties with economic benefits for farmers and nutritional value for consumers. The major challenges for agriculture production are global climatic changes and their associated issues [5]. The ability of crops to adapt to biotic and abiotic stresses is crucial for selecting breeding materials. So, plant breeding provides an opportunity to integrate desirable traits and develop new crop varieties (Fig. **2**). Breeding highly adopted and better-performing crop varieties are fundamental to increasing agricultural productivity. Releasing the new varieties usually evaluates their performance under various environments to select reliable performers [15]. Now, plant breeding looks more promising. Many novel breeding techniques are being used along with good knowledge and tools to ensure the advancement in plant breeding. Varshney *et al.* [16] proposed the 5G breeding approaches for future crop improvement. These are 1) genome, 2) germplasm, 3) genes, 4) genomic breeding, and 5) genome editing [16]. The importance of this 5G approach has been highlighted by many researchers [16-18]. It is predicted that these 5Gs may collectively play a crucial role in precision breeding in the future. Ignorance of one of them may interfere with the efficiency of breeding. Researchers have developed improved crop varieties through precession breeding, which includes the presence of 5G approaches. The 5G is crucial and is the backbone of future crop improvement. These approaches require better technical facilities and advanced technologies.

**Fig. (3):**
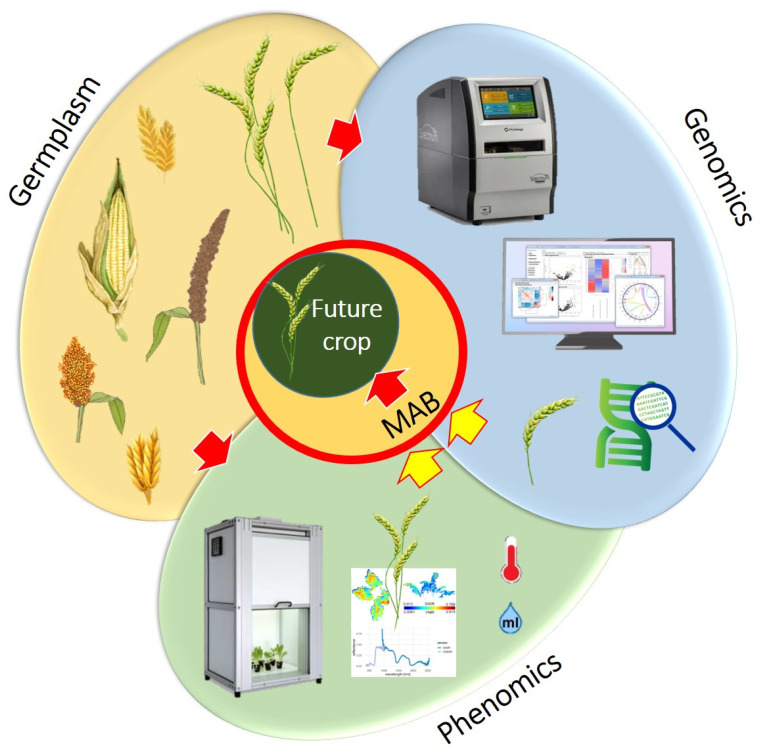
Phenomic and genomic approaches for precision breeding. This diagram describes the phenomic and genomic approaches for developing future crops.

Conventional and other breeding techniques help accomplish efficient breeding (Table **1**). Plant breeders and agriculture scientists worldwide use new techniques such as speed breeding, genome editing, and high throughput phenotyping facilities for crop improvement programs [18-20]. The genome-assisted tool helps for increasing the efficiency of crop breeding. Also, high-throughput phenomic facilities improve the efficiency of plant breeding. Nowadays, many promising tools are available in phenomics. Both genomic and phenomic approaches are crucial for precision breeding (Fig. **3**) and have significantly contributed to plant breeding. Plant breeding has many scopes and objectives; its final goal is crop improvement. So, properly applying plant breeding with sophisticated tools can help increase food production and reduce food demand in the future.

### 2.1 Phenomics

Plant phenotyping is necessary to develop a new crop variety [19]. The phenotyping is still challenging; it is non-uniform and environmentally sensitive [21]. Traditional phenotyping relying on manual measurement is laborious, time-consuming, cause fallible data, and is expensive [19]. False selection of breeding material through phenotypic selection significantly affects the introgression of desirable traits/genes in the new varieties. Finally, the developed varieties may fail to perform the selection criteria under the field trial. It causes time and economic losses, and especially, it could lead to the narrowing of the gene pool and reduce the efficiency of future crop improvement. The phenotyping of germplasm is a crucial step for an efficient crop improvement program. Phenotype-based crop selection is essential for GS. It is one of the approaches for 5G. Many advanced phenomic facilities are now available for efficient phenotyping. Advance phenomics is a combination of different steps; it starts with 1) determination of the desired trait, 2) accurate data collection from high-throughput devices, and 3) calculation and validation of results [22]. The first step is done with the help of advanced phenotypic tools. The second and third steps depend on computing methods using software [23]. It leads to digital phenomic studies and will enhance plant trait measurements' capability, speed, coverage, repeatability, accuracy, and cost-effectiveness [24, 25]. The availability of advanced facilities and automation in phenomics will benefit phenotypic selection. It provides a viable solution to large-scale phenotyping data collection and processing during phenotypic selection. Large-scale phenotyping under controlled environmental conditions is very helpful and accurate during any time of data collection. High-throughput phenomics facilities will streng- then the phenomics approaches for efficient crop improvement in the coming years.

Phenotyping by robots and the availability of automation emerged as a high-throughput technology to accurately measure plant (morphological, chemical, and physiological) traits. Different sensors are attached to ground-based vehicles and used for plant phenotyping [26]. Initially, the high-throughput phenomic platforms were expensive in construction and maintenance. It also demanded technical experts to control the automation system during the data collection. High-throughput phenomic technology strengthens the phenotypic study and improves the efficiency of crop breeding. Robotic phenotyping has the potential to effectively monitor changes in crop behavior over time in controlled environments and field conditions [26, 27]. Utilizing high-throughput phenomic technology provides uniformity of scientific data at any time [5]. Many robotic systems have been developed for phenotyping and successfully applied in crops (Table **2**). For example, advanced automated plant transport and imaging systems (thermal imager) were used to determine the drought tolerance in interbreed lines of maize under a controlled growth chamber [22]. Vinobot (autonomous ground vehicle) and vinoculer (mobile observation tower) are two robotic systems used for high-throughput field phenotyping. This robotic system is a 3D imaging sensor mounted on a mobile platform to measure plant height and leaf area index [28]. Therefore, the 3D imaging sensor provides accurate phenotypic data from field conditions. The autonomous robot (BoniRob) platform is used to measure the plant height, stem thickness, and biomass under field conditions [29]. Field-based phenotyping platforms are very effective for crop phenotyping at the individual and field-plot scales. It has both advantages and disadvantages during the phenotypic data collection. For example, the “Field Scanalyzer” is a rail-based gantry system for field phenotyping of crops [29]. This equipment has a ∼300 kg sensor array. It includes a visible camera, 3D laser scanner, visible to near-infrared hyperspectral camera, thermal infrared camera, four-channel amplified radiometer, CO_2_ sensor, and chlorophyll fluorescence sensor. This equipment (fully automated) can be used to measure the canopy development of all crop growth stages in field conditions [29]. Field Scanalyzer-equipped sensors are beneficial in providing accurate data from the field. The main limitation of rail-based systems are 1) covering a limited area, 2) difficulty in using marsh area, 3) high cost, 4) maintenance 5) difficulty to transport. The sensors mounted on manually operated vehicles or self-propelled tractors may resolve many of these issues. The technologies have grown day by day. It can help to upgrade the phenotypic platform and give more accurate data in the future. Many researchers have reviewed the robotic and associated technology in agriculture and its scope for high-throughput plant phenotyping [19, 30-33]. Imaging, sensor devices, and robotic systems are helpful for plant phenotyping in different ways. Plant breeders should utilize high-throughput phenomic platforms for the phenotypic selection of crops in the future. Plant breeders need to follow the digital phenotyping strategy in the coming decades. It will accelerate the efficiency of crop breeding.

### 2.2 Genome Sequencing

The application of sequence technologies has led to notable advances in whole-genome sequencing. As a result, tremendous progress has been made in crop improvement programs in the last few years. The advancement in sequence technology helps understand the genome organization of many crops [34]. Identifying the genetic variation underlying phenotypic changes is crucial for understanding the wide variety of biological processes. The NGS technology opens the scope to understand better the genetic basis of variation of phenotypes with high-resolution genomic data [35]. Many types of DNA-sequencing technologies have been developed so far. Several researchers have reviewed the scope of DNA sequence technology in plant breeding [36-38]. These approaches have made it possible to identify QTL/genes for multiple traits and their precise transfer into the elite background. The help of GBS and genomic-assisted tools have enhanced the precision in conventional crop breeding programs. Many reports are available on developing new cultivars/varieties with enhanced tolerance /resistance to biotic and abiotic stresses through MAS [39]. Genome sequencing technologies also help crop improvement in different ways. Crop breeding advances, such as mutation breeding, MAB, and genome editing, are very effective for crop improvement [40, 41]. Sequencing technologies help detect the mutation/variation of targeted crops within a short period at a low cost. It helps to develop new varieties quickly compared to conventional breeding. There have been many outstanding achievements in sequencing technology. This led to the availability of the whole-genome sequence of many cereal and non-cereal crops. The availability of DNA sequencing information helps discover novel genes and molecular markers linked with important agronomic traits and provides an opportunity for crop improvement. The DNA sequencing platform also offered the opportunity to find novel single nucleotide polymorphism (SNP) markers. The SNP marker help to detect the single nucleotide variation among genotypes, which is very helpful for constructing a genetic map for efficient crop improvements. Sequence technology seems to be the foundation for advanced breeding techniques for efficient crop improvement. Researchers have already developed new crop varieties using advanced breeding technologies with the help of genome sequence platforms [11, 42-44]. Advanced sequence technology influenced plant breeding and changed it from conventional breeding to GAB.

## 3 CONVENTIONAL BREEDING TO GENOMICS-ASSISTED BREEDING FOR CROP IMPROVEMENT

Crop improvement was based on phenotypic selection through conventional breeding during the past decade. Phenotypic-based selection is not much effective for crop improvement. Conventional breeding involves hybridization between two parents (genetically diverse) and subsequent selection over different generations to develop high-yielding crop variety. This technique effectively improves crop performance and provides a safer tool [45, 46]. But, this approach has some limitations. These are 1) it requires 12-15 years to develop new crop variety, 2) high cost and lack of high throughput phenotypic tools, 3) the influence of high environmental noise for phenotypic selection, and 4) less effectiveness for complex and low heritable traits [47]. Selection criteria for breeding material/improved variety have been challenging for each crop in the past decades. The plant breeders struggle with selecting good breeding materials for crop improvement. Reducing the release time of new crop varieties is the main objective of speed breeding. This can be accomplished through various technologies and platforms such as high throughput phenotyping, MAS, GBS, GS, and genome-editing, *etc*. For example, the automated high throughput phenomic facilities strengthen the phenotypic selection for speed breeding. The advent of molecular and genomic approaches has allowed researchers to track the specific genes known to influence traits of interest [48]. As a result, scientists have used MAS for GS to develop new crop varieties within a short period. It is a foundation of the genotype-based selection of individuals for crop breeding. The MAB approaches have helped enhance crops' stress (biotic and abiotic) adaptation. The advanced sequencing and phenomics platforms have transferred MAB to GAB. The GAB provides an efficient crop improvement strategy and accelerates the breeding works.

### 3.1 Past and Present Progress in Plant Breeding

Traits-specific germplasm evaluation is crucial for identifying the best breeding materials for desirable traits [49]. The genetic improvement of crops during the past century was based on the phenotypic selection method. It is not much effective for crop breeding. In crops, the quantitative traits are governed by QTL [50]. The crop genetic mapping and molecular characterization of valuable QTL facilitate MAB in crop improvement [51]. The genetic (association/linkage) mapping is crucial for identifying the genetic basis of quantitative traits in crops. Different types of molecular markers were developed from the genome of many crops [52-56]. The molecular marker (DNA-based) can identify the allelic variation in the valuable genes underlying important agronomic traits. The use of molecular markers can enhance the efficiency and preciousness of breeding. Efficient GS offers the opportunity to improve the genetic pool and increase crop production. The availability of genome-wide molecular markers has made the use of GS in crops possible. The GS is a method to predict the genomic value of breeding materials based on the genomic estimated breeding value (GEBV) [57]. The first-generation molecular markers such as random amplified polymorphic DNA (RAPD) [58-60], interspersed simple sequence repeat (ISSR) [61, 62], sequence characterized amplified region (SCAR) [63, 64], single primer amplification reaction (SPAR) [65, 66], simple sequence repeat (SSR) [67-69] have been used successfully to determine the genetic basis of phenotypic variation in crops. These molecular markers could be helpful for the genetic basis of GS for crop improvement.

The DNA-based molecular marker will increase the efficiency and precision of conventional breeding *via* MAS [70]. The molecular markers are helpful for alleles/QTL/gene identification. Markers are linked with traits of interest to select crops with desirable alleles/genes affecting the target trait. Many QTL have been identified in various crops using first-generation molecular markers. For example, QTL associated with agronomically important traits were identified in rice [71-74], maize [75-77], barely [78-81], wheat [82-84], sorghum [85-87], and small millets [88-90] under various biotic and abiotic stress conditions. Identified markers are associated with valuable QTL/genes that can help improve crop yields through precision breeding. First-generation molecular markers were helpful for the rapid identification of useful QTLs related to stress tolerance. Molecular markers provide the foundation of the GAB program. The first-generation molecular markers have some drawbacks; it affects the GS for efficient crop breeding. Locus-specific and co-dominant markers are more suitable than other dominant markers for MAS. The dominant molecular markers may lead to inefficient crop improvement GS due to their low reproducibility and reliability. The NGS-based high-throughput molecular markers may resolve these issues.

**Fig. (4):**
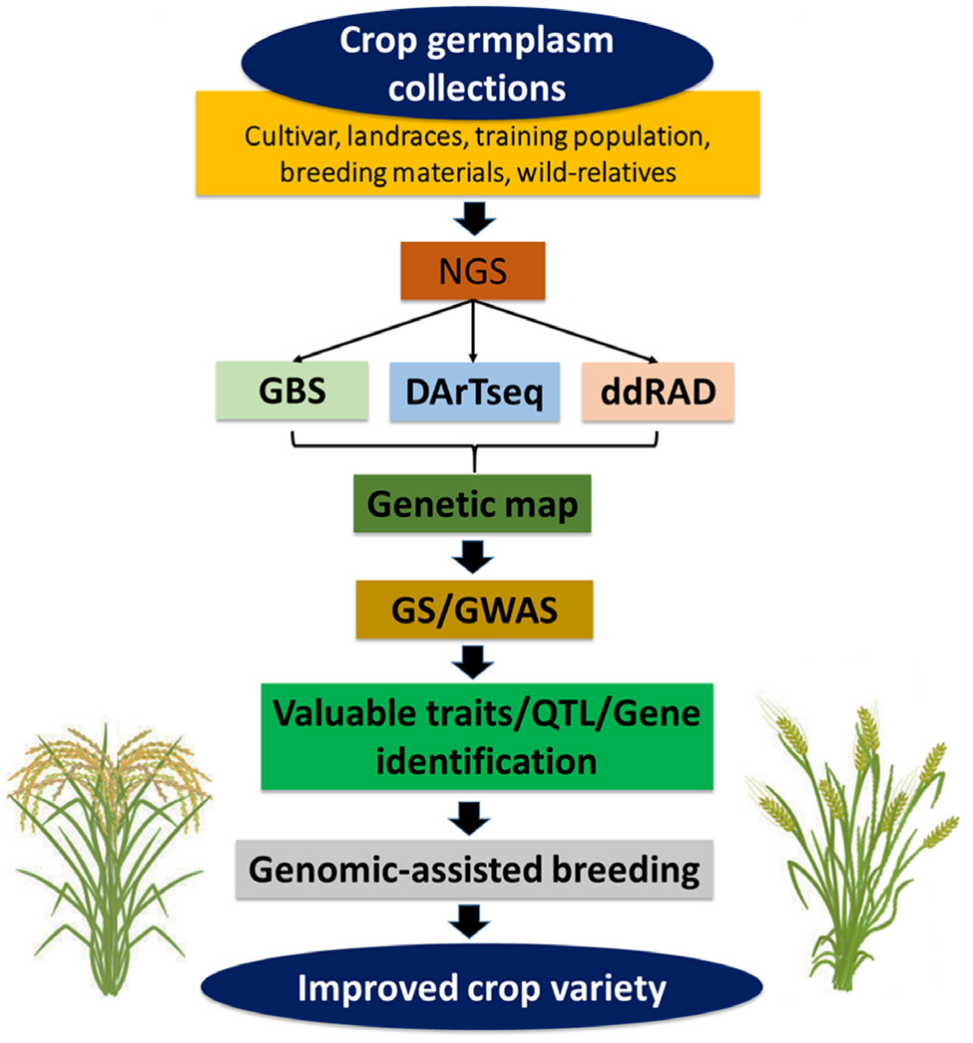
Role of genome sequence technology in crop improvement. The NGS provides an opportunity for high-throughput genotypic data, which could help detect high-throughput molecular markers, speed up MAS approaches, and, finally, be helpful for MAB.

NGS provides an opportunity for high-throughput genotyping by sequencing (GBS) technology, which could help to detect high-throughput molecular markers and speed up MAS approaches (Fig. **4**). Several researchers have successfully employed genome-wide association studies (GWAS) by GBS. It is helpful for GS in crop breeding. Most notably, an advanced genotyping system helps find the SNP, which was used to discover valuable QTL/genes in many crops. Many GWASs are available in major crops such as rice, maize, and wheat compared to other crop plants (Table **3**). For example, by GBS technology, Spindel *et al.* [91] discovered QTL governing grain yield and flowering time in elite breeding lines of rice. This study identified 73,147 novel SNP markers. Huang *et al.* [92] evaluated agro-morphological traits of 517 landraces population of rice by GBS and identified valuable QTL for tiller number, grain width and length, spikelet number, gelatinization temperature, amylose content, apiculus color, pericarp color, hull color, heading date, drought tolerance and degree of seed shattering. GWAS for agro-morphological and other valuable traits have also been reported in many crops, such as rice, maize, wheat, barley, sorghum, pearl millet, and small millets. The GWAS is useful for GS for crop improvement. Advanced GS methods help in the development of new cultivars through MAB. So, GWAS provides the opportunity for precision breeding. The advances in phenomics and genomic platforms allow GS for efficient crop improvement. It also helps in the development of advanced breeding techniques for crop improvement. The high-throughput genomic and phenomic facilities are the two pillars of precision breeding supporting crop improvement.

## CONCLUSION AND FUTURE PROSPECTS

Plant breeding has made a significant contribution to the increase in agricultural productions. Due to many challenges in conventional breeding, the plant breeders are incorporating more efficient strategies in crop breeding programs to improve crops. The availability of high-throughput phenomic and genomic facilities has effectively improved crop breeding. The advanced phenomic approaches have provided high-throughput phenotypic data and strengthened the phenotypic selection for crop breeding. Molecular marker technologies notably contribute to the shift from conventional breeding to GAB. It is successfully implemented in GS for crop breeding. Tremendous advances in genome sequence technology have transformed MAB into GAB. The rapid advancement in phenomics and genomic approaches has helped precision breeding. It provides the development of new cultivars in a short period compared to conventional breeding. Plant breeders need to pay more attention to crop improvement through GAB. GAB could help to improve crop production and strengthen food security in developing countries.

## Figures and Tables

**Table 1:** Features of types of breeding techniques used for plant breeding and crop improvement.

**Types of Breeding**	**Features**
Cross breeding	Introducing desirable traits from suitable parental genomes Required back crossing for derived population Transgene-free crop plants Required 12-15 years for variety releasing
Mutation breeding	Random mutagenesis Required back cross Transgene-free crop plants Required 8-10 years for variety releasing
Genome-assisted breeding	Random mutagenesis Required back crossing for derived population Transgene-free crop plants Required 5-8 years for variety releasing
Transgenetic breeding	Introducing desirable traits from an organism Foreign DNA is integrated Transgene crop plants Required 3-4 years for variety releasing
Breeding by genome editing	Targeted mutagenesis Back cross is not required Transgene-free crop plants Required 2-5 years for variety releasing

**Table 2:** Plant, technology, equipment, phenotypic traits, and working platform for high-throughput phenomics facilities used for plant phenotyping, with associated reference.

**Name of the Plant**	**Technology Used**	**Name of the Equipment**	**Plant Phenotypic Traits**	**Condition/Platform**	**References**
*Arabidopsis*	Imaging	Growscreen Fluoro	Plant growth and chlorophyll fluorescence	Controlled condition	[93]
	Camera	Sony SSC-DC393P	Plant leaf growth	Controlled condition	[94]
Maize	Sensors	Spectroradiometer	Drought tolerance	Controlled condition	[95]
	Sensors	Chlorophyll meter	Chlorophyll content	Controlled condition	[95]
	Sensors imaging	-	Plant leaf area	Field condition	[96]
	Hyperspectral imaging	Phenovision	Drought tolerance	Controlled condition	[97]
	Robotic system	TerraSentia	Corn stand counting	Field condition	[98]
	Robotic system	Phenomobile	Plant height	Field condition	[99]
	Robot imaging system	-	Plant height, leaf angle, plant orientation, and stem diameter	Field condition	[100]
	Robotic system	BoniRob	Plant height, stem thickness and biomass	Field condition	[29]
	Robotic imaging	-	Stem position and plant height	Field condition	[101]
	Robot imaging system	Vinobot	Plant height and leaf area index	Field condition	[28]
	Robot imaging system	Vinoculer	Plant height and leaf area index	Field condition	[28]
Rice	Sensors imaging	Field Servers	Determination of rice bugs	Field condition	[102]
	Thermal imaging	PlantScreen	Drought tolerance	Controlled condition	[103]
Wheat	Hydraulic push press	Proxy Screen	Root depth and distribution	Field condition	[104]
Cotton	Sensor	LeeAgra 3434 DL	Measurement of canopy height	Field condition	[105]
Sorghum	Sensor imaging system	-	Plant height	Field condition	[106]
	Robotic stereo imaging	TERRA-MEPP	Plant height and stem width	Controlled condition	[98]
	Robot system	-	Leaf area, leaf length and width	Filed condition	[107]
	Robot imaging system	Vinobot	Plant height and leaf area index	Field condition	[28]
	Robot imaging system	Vinoculer	Plant height and leaf area index	Field condition	[28]
	Robotic system	Robotanist	Stalk strength measurement	Field condition	[26]

**Table 3:** Genomic selection (GS) is applied for various traits in major crops using next-generation sequencing (NGS) technology. The details such as the name of the crop, sequence platform used for genome-wide association studies (GWAS), type of population, size of the population, name of trait, and the total number of the identified marker are provided with references.

**Crop Name**	**Sequencing Platform**	**Type of Population**	**Population Size**	**Traits**	**Total Number of SNP Markers Identified**	**References**
Rice	GBS	Elite breeding lines	364	Grain yield and flowering time	73147	[91]
	DArTseq	Training population	343	Grain yield and plant height	8336	[108]
	GBS	Landraces population	517	Agronomic traits	~3.6 million	[92]
	GBS	Landrace and elite	529	Agronomic traits	4,358,600	[109]
	GBS	Mini core collection	529	Mineral elements	∼6.4 million	[110]
Maize	GBS	Biparental populations	3273	Drought stress	58,731	[111]
	GBS	Doubled-haploid lines	504	Grain yield, anthesis date anthesis-silking interval	1,58,281	[112]
	GBS	Inbred lines	296	Grain yield, anthesis date anthesis-silking interval	2,35,265	[112]
	DArTseq	Mini core collection	238	Ear rot disease resistance	23.154 DArTseq markers	[113]
Wheat	GBS	CIMMYT breeding lines	365	Stem rust resistance	4,040	[114]
	GBS	CIMMYT breeding lines	254	Grain yield	41,371	[7]
	GBS	Inbred lines	1477	Grain yield	81,999	[115]
	GBS	Inbred lines	1127	Grain yield and yield-related traits, and protein content	38,893	[116]
	GBS	Breeding lines	273	Fusarium head blight resistance	19,992	[117]

## LIST OF ABBREVIATIONS

GAB: Genomics-Assisted Breeding
GBS: Genotyping by Sequencing
GEBV: Genomic Estimated Breeding Value
GS: Genome-Wide Selection
GWAS: Genome-Wide Association Study
ISSR: Inter-Simple Sequence Repeat
MAS: Marker-Assisted Selection
NGS: Next-Generation Sequencing
QTL: Quantitative Trait Loci
SCAR: Sequence Characterized Amplified Region
SNP: Single Nucleotide Polymorphism
SPAR: Single Primer Amplification Reaction
SSR: Simple Sequence Repeat
WGAS: Whole-Genome Association Study
